# White matter integrity as a mediator between socioeconomic status and executive function

**DOI:** 10.3389/fnhum.2022.1021857

**Published:** 2022-11-18

**Authors:** Danielle Shaked, Leslie I. Katzel, Christos Davatzikos, Rao P. Gullapalli, Stephen L. Seliger, Guray Erus, Michele K. Evans, Alan B. Zonderman, Shari R. Waldstein

**Affiliations:** ^1^Department of Psychology, University of Maryland, Baltimore County, Baltimore, MD, United States; ^2^Laboratory of Epidemiology and Population Sciences, National Institute on Aging Intramural Research Program, Baltimore, MD, United States; ^3^Department of Psychology, VA Boston Health Care System, Boston, MA, United States; ^4^Division of Gerontology, Geriatrics, and Palliative Medicine, Department of Medicine, University of Maryland School of Medicine, Baltimore, MD, United States; ^5^Geriatric Research Education and Clinical Center, Baltimore VA Medical Center, Baltimore, MD, United States; ^6^Artificial Intelligence in Biomedical Imaging Laboratory (AIBIL), Perelman School of Medicine, University of Pennsylvania, Philadelphia, PA, United States; ^7^Department of Diagnostic Radiology, University of Maryland School of Medicine, Baltimore, MD, United States; ^8^Division of Nephrology, Department of Medicine, University of Maryland School of Medicine, Baltimore, MD, United States

**Keywords:** executive function, health disparities, diffusion tensor imaging, white matter integrity, socioeconomic status, neuroanatomical health, fractional anisotropy

## Abstract

**Introduction:**

Lower socioeconomic status (SES) is associated with poorer executive function, but the neural mechanisms of this association remain unclear. As healthy brain communication is essential to our cognitive abilities, white matter integrity may be key to understanding socioeconomic disparities.

**Methods:**

Participants were 201 African American and White adults (ages 33–72) from the Healthy Aging in Neighborhoods of Diversity across the Life Span (HANDLS) SCAN study. Diffusion tensor imaging was used to estimate regional fractional anisotropy as a measure of white matter integrity. Adjusting for age, analyses examined if integrity of the anterior limb of the internal capsule (ALIC), external capsule (EC), superior longitudinal fasciculus (SLF), and cingulum mediated SES-executive function relations.

**Results:**

Lower SES was related to poorer cognitive performance and white matter integrity. Lower Trails B performance was related to poorer integrity of the ALIC, EC, and SLF, and lower Stroop performance was associated with poorer integrity of the ALIC and EC. ALIC mediated the SES-Trails B relation, and EC mediated the SES-Trails B and SES-Stroop relations. Sensitivity analyses revealed that (1) adjustment for race rendered the EC mediations non-significant, (2) when using poverty status and continuous education as predictors, results were largely the same, (3) at least some of the study’s findings may generalize to processing speed, (4) mediations are not age-dependent in our sample, and (5) more research is needed to understand the role of cardiovascular risk factors in these models.

**Discussion:**

Findings demonstrate that poorer white matter integrity helps explain SES disparities in executive function and highlight the need for further clarification of the biopsychosocial mechanisms of the SES-cognition association.

## Introduction

As the most complex cognitive domain, executive function is essential for successfully navigating through our everyday lives (Marcotte and Grant, 2010). Lower levels of socioeconomic status (SES) have been associated with lower levels of executive function (Farah, 2017). This may be due, at least partly, to the protracted maturation of brain areas and circuits linked to executive function, making them more susceptible to environmental influence (Giedd, 2004; Blakemore and Choudhury, 2006).

Many behavioral and psychosocial factors have been hypothesized to contribute to the SES-executive function relation, including stress, access to resources, cognitive stimulation, health behaviors, and environmental exposures (Adler and Ostrove, 1999; Baum et al., 1999; Miller et al., 2011; Shonkoff et al., 2012). The interrelated, biological mechanisms responsible for the association between SES and executive function are less understood, although some studies have shown that volumetric reductions in relevant brain regions contribute (Marcus Jenkins et al., 2013; Shaked et al., 2018). Less is known about the contribution of white matter integrity.

Diffusion tensor imaging (DTI) studies suggest that communicative white matter pathways are an important component of the neurostructural foundation of our cognitive abilities (Kraus et al., 2007; Voineskos et al., 2012). Declines in fractional anisotropy (FA)–used to measure microstructural properties including integrity (Alexander et al., 2007)–relates to poorer executive function (Grieve et al., 2007).

Consistent throughout the DTI literature, studies have identified several white matter tracts that, independent of age, relate to executive function. Four of these tracts are the superior longitudinal fasciculus (SLF), anterior limb of the internal capsule (ALIC), cingulum, and external capsule (EC).

The SLF is an associative bundle composed of two parallel fiber pathways that connect the temporal, parietal, and frontal regions (Martino et al., 2013). More specifically, it connects the middle frontal gyrus/dorsolateral prefrontal cortex, one of the primary brain regions responsible for our executive functions, with various other parts of the brain (Stuss and Knight, 2002). It has been linked to executive function in numerous studies (Charlton et al., 2010; Konrad et al., 2010; Burzynska et al., 2011; Vestergaard et al., 2011; Noble et al., 2013), with certain studies demonstrating a direct link to the Stroop test (Sasson et al., 2012, 2013; Jacobs et al., 2013).

The ALIC contains the anterior thalamic radiation, which connects the medial and anterior thalamic nuclei with the prefrontal cortex and the cingulate gyrus (Zhou et al., 2003), and is primary to higher-order functions (Schmahmann and Pandya, 2008). Given its location, it appears to be important for executive function (Catani and Thiebaut de Schotten, 2008; Schmahmann and Pandya, 2008; Konrad et al., 2010; Rae et al., 2012), and has been specifically linked to performance on the Stroop (Wolf et al., 2014), Trails B (Jacobs et al., 2013), and a non-verbal span task (Kennedy and Raz, 2009).

The cingulum is a medial associative bundle that runs within the cingulated gyrus all around the corpus callosum (Catani and Thiebaut de Schotten, 2008). Its longest fiber runs from the anterior temporal gyrus to the orbitofrontal cortex, and its shorter fibers connect the medial frontal, parietal, occipital, and temporal lobes (Catani and Thiebaut de Schotten, 2008). The cingulum is part of the corticolimbic circuit and is involved in attention, memory, and emotions (Rudrauf et al., 2008). It has also been directly associated with executive function ability (Murphy et al., 2007; Skranes et al., 2009; Konrad et al., 2010; Schermuly et al., 2010; Kantarci et al., 2011).

The EC is a bundle of cholinergic, associative fibers that connect from the basal forebrain to the frontal, temporal, and parietal lobes (Selden et al., 1998). It is thought to be important for executive functions, cognitive control, and emotion regulation (Korgaonkar et al., 2011; Xiao et al., 2015). While less research has focused on the EC compared to other associative tracts, potentially due to resolution difficulties in identifying this tract (Mori and Crain, 2005; Kraus et al., 2007), studies have related executive function performance with white matter integrity of this tract (Mabbott et al., 2006; Kraus et al., 2007), as well as with hyperintensities in this region (O’Brien et al., 2002).

The limited available research examining the relation between SES and white matter integrity is equivocal. Studies have shown significant relations diffusely throughout the brain (Shaked et al., 2019a) and in specific tracts, particularly the SLF and cingulum (Noble et al., 2013; Ursache and Noble, 2016). However, at least one study found no association between white matter integrity and SES (Jednorog et al., 2012). Another investigation similarly found no relation between SES and FA, but noted a significant gene by SES interaction wherein higher SES was associated with higher heritability of FA (Chiang et al., 2011). Little is known regarding the mechanisms by which disadvantaged SES may relate to white matter integrity, but the aforementioned psychosocial factors (e.g., stress) may contribute given their well-established link to socioeconomic differences in gray matter structure and function (McEwen and Gianaros, 2010).

Taken together, there are relations among SES, executive function, and neuroanatomical endpoints, although more research is needed to clarify the relation between SES and white matter integrity. Additionally, while researchers have begun to explore the volumetric underpinnings of the SES-executive function relations, no studies have investigated whether white matter integrity mediates these associations. We therefore examined whether socioeconomically-linked executive function decrements are mediated by lesser white matter integrity. This work is essential considering the public health significance of weakened executive functions, the disparities in executive function among SES groups, and the limited understanding as to how and why these relations persist.

## Materials and methods

### Sample and participants

Participants were drawn from the Healthy Aging in Neighborhoods of Diversity across the Life Span (HANDLS) SCAN study, an investigation of brain health disparities (Waldstein et al., 2017). HANDLS SCAN is an ancillary study of the larger HANDLS investigation, a prospective, epidemiologic study aimed at understanding health disparities across a socioeconomically diverse group of African American and White community-dwelling adults in Baltimore, MD (Evans et al., 2010). Participants were recruited from HANDLS to take part in HANDLS SCAN, which obtained MRI data from HANDLS participants who completed their first or second complete follow-up visit.

Healthy aging in neighborhoods of diversity across the life span (HANDLS) study inclusions were (1) age 30–64 at baseline; (2) able to provide informed consent; (3) able to complete at least five of the nine tests given on the Mobile Research Vehicle (MRV); (4) have valid picture identification. HANDLS SCAN participants had the following additional exclusions: history of dementia, stroke, transient ischemic attack, or other neurological disorder, or carotid endarterectomy; MRI contraindications (e.g., claustrophobia); diagnosis of a terminal illness; HIV positive status. In addition to being asked about dementia history, all participants completed a Mini-Mental State Examination. Only those who received a score of 24 or higher were included in the study.

Out of the 2,468 actively enrolled HANDLS participants, 252 participants enrolled in HANDLS SCAN and successfully completed neuroimaging. 21 participants were excluded because their MRI scans yielded incidental clinical findings, and 30 were excluded due to missing data on key study variables, yielding 201 participants for the present analysis. Compared to the larger HANDLS sample, our imaging subsample was more likely to be White (*p* < 0.001), above the poverty line (*p* < 0.05), and younger (*p* < 0.05). There was no significant sex difference between our imaging subsample and the overall sample.

### Procedure

#### Healthy aging in neighborhoods of diversity across the life span

Participants were recruited from 13 Baltimore neighborhoods pre-determined to yield a wide distribution of sociodemographic characteristics. Successfully recruited and consented individuals were asked to complete a household survey inquiring about demographic, psychosocial, and physiological information. In an additional appointment on the MRV, participants underwent a comprehensive cognitive battery and other procedures not relevant here. The Institutional Review Board (IRB) of the National Institutes of Health approved the HANDLS study. Research was conducted in compliance with the Helsinki Declaration.

#### Healthy aging in neighborhoods of diversity across the life span scan

Eligible HANDLS participants were approached during their MRV visit and invited to participate in HANDLS SCAN. Those who expressed interest were given an MRI eligibility screener and provided written informed and HIPAA consent. They were then examined by a physician at the University of Maryland General Clinical Research Center for a brief medical evaluation to identify any acute medical problems since their last HANDLS visit, re-administer the MRI eligibility checklist, review current medications and assess whether there were any contraindications to the performance of HANDLS SCAN testing. Participants underwent MRI acquisition in the Department of Diagnostic Radiology and Nuclear Medicine at the University of Maryland School of Medicine. The IRBs of the University of Maryland, Baltimore and the University of Maryland, Baltimore approved this study. Participants received $50 for their participation. The time between cognitive data collection and imaging was an average of 0.52 years.

### Measures

#### Sociodemographic variables

The SES index was comprised of education and poverty status, with both variables dichotomized. Below median education (0 = 12 years or above; 1 ≤ 12 years) and income below 125% of the 2004 federal poverty line relative to family size and household income (0 = above the poverty line; 1 = below the poverty line) was considered low SES. Participants who met neither of those criteria were labeled as high SES. Additional demographic variables were biological sex (0 = female; 1 = male), age (years), and self-identified race (0 = White; 1 = African American). As racial categories are weak proxies for biological and genetic differences (Krieger, 2000), HANDLS conceptualizes race as a social construct that is impacted by sociopolitical factors.

#### Tests of executive function

##### Trail making test

The Trails Making Test is a test of attention, processing speed, sequencing, mental flexibility, visual search, and motor functioning (Spreen and Strauss, 1998). The test involves two parts. Part A is a measure of processing speed among other abilities. Part B is a more complex task, requiring a higher level of visual-perceptual processing and set-switching, and as such, is more often used as a test of executive function. Trails B was used as the primary outcome variable and Trails A was used for exploratory analyses. While the normative time cutoff for Trails B is 300 s (Strauss et al., 2006), we extended the cutoff to 600 s to allow for greater variability in task performance.

##### Digit span backwards

WAIS-R’s Digit Span Backwards measures attention, concentration, and working memory (Lezak, 1995). The outcome variable is the total raw score, which is calculated as the number of digits repeated, backwards, accurately.

##### Stroop color and word test

The Stroop is a measure of cognitive control and inhibition (Strauss et al., 2006). The test consists of three separate trials. Trial 1, Stroop Words, and Trial 2, Stroop Colors, capture processing speed. Trial 3 has an inhibition component, thus making it a strong test of executive function. The primary outcome variable is the number of items correctly read in 45 s for Trial 3, Stroop Inhibition. Trial 1 was used for exploratory analyses.

### Rationale for covariates

Diffusion tensor imaging (DTI) measures (Hugenschmidt et al., 2008; Giorgio et al., 2010) and cognition (Madden et al., 2012) are sensitive to age-related decline. Thus, age was an adjustment variable in all analyses. The literature on sex-related differences in white matter is mixed (Gilmore et al., 2018). Therefore, sex was not a primary covariate, but included in exploratory analyses. Racial differences in white matter are largely unexamined. However, utilizing the same participant sample, SES variable, and DTI data, we recently found no racial differences in FA across the brain’s four lobes (Shaked et al., 2019a). Nonetheless, racial disparities in global and regional brain volume, and white matter lesion volume have been found in the HANDLS sample (Waldstein et al., 2017; Shaked et al., 2019b). Exploratory analyses therefore included race.

Given the role that cardiovascular risk plays in brain disparities, sensitivity analyses covaried for body mass index (BMI), diabetes, and hypertension. BMI was computed as weight divided by height squared (kg/m^2^). Hypertension and diabetes were dichotomous variables (coded as 0 = absent, 1 = present). Hypertension was evaluated based off self-reported history, use of anti-hypertensive medications, or resting systolic or diastolic pressures > 140 mm Hg or > 90 mm Hg, respectively. Diabetes was determined by a fasting blood glucose level of > 126 mg/dl, self-reported history, or use of relevant medications.

### Magnetic resonance imaging

Cranial magnetic resonance images were acquired using a Siemens Tim-Trio 3.0 Tesla scanner at the Department of Diagnostic Radiology at University of Maryland Baltimore’s School of Medicine. In addition to the standard brain imaging protocol, which includes axial T1, T2, FLAIR images, a high-resolution axial T1-weighted MPRAGE covering the entire brain was acquired. It was used as an anatomic reference and to extract structural parameters.

Diffusion tensor imaging (DTI) images were acquired using a multi-band spin echo EPI sequence with an in-plane isotropic resolution of 2 × 2 mm, and 2 mm slice thickness over a 22.4 cm field of view. A total of 66 slices were acquired at a TE = 122 ms, TR = 3,300 ms, and flip angle = 90°. Diffusion weighting scheme was a 2-shell (*b* = 1,000, 2,500), optimized for uniform sampling of each shell and non-overlapping diffusion directions of 60 and 120 for each shell, respectively, and 6 b0 volumes. Image acquisition time was 10 min. Preprocessing of the raw diffusion weighted imaging data involved motion correction using FSL’ s “eddycorrect” tool (Andersson and Sotiropoulos, 2016) and de-noising using the Joint Linear Minimum Mean Squared Error software (jLMMSE; Tristán-Vega and Aja-Fernández, 2010). Multivariate line fitting was used to reconstruct the DTI images by fitting the de-noised diffusion weighted imaging data (Pierpaoli and Basser, 1996). Fractional anisotropy (FA) maps are computed from the DTI images using an in-house software package (select components were taken from the following software package: https://github.com/DiCIPHR-Lab/Fernet). Specifically, the values were derived from the variance of the average of the three eigenvalues of the diffusion tensor at each voxel, to measure the degree of anisotropy of the diffusion at the voxel level. FA values quantify the degree of water diffusion directionality within the brain tissues, and are used as a measure of regional white matter integrity, with higher FA values indicating healthier white matter integrity. To calculate regional average FA values, FA maps) were first aligned to subject’s T1-weighted scan using ANTs (Avants et al., 2009). DTI-specific regions of interest were segmented on the T1-weighted scan *via* deformable registration of the JHU-MNI-ss atlas, or “Eve,” (Oishi et al., 2009) using ANTs deformable registration (Avants et al., 2009).

### Data analyses

**FIGURE 1:**
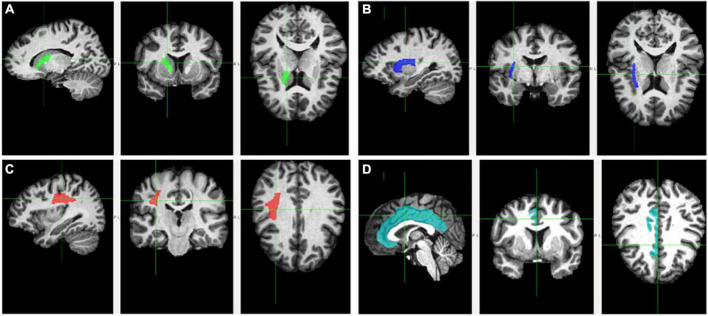
Fractional anisotropy of the examined white matter tracts. Tracts are delineated by the cross and respective color. Tracts are depicted on sagittal (left), coronal (middle), and axial (right) planes. **(A)** Anterior limb of the interior capsule (green); **(B)** external capsule (blue); **(C)** superior longitudinal fasciculus (red); and **(D)** cingulum (teal).

**FIGURE 2:**
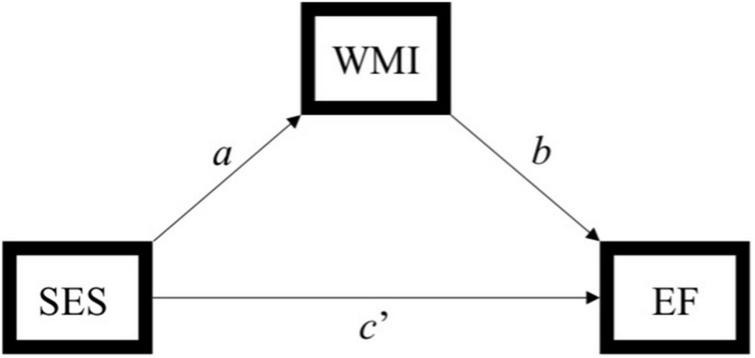
Simplified path model used to assess the indirect effect of socioeconomic status (SES) on executive function through white matter integrity. Indirect effect of SES on executive function through white matter integrity = *ab*. SES, socioeconomic status; WMI, white matter integrity; EF, executive function. Hayes (2013) model 4.

Statistical analyses were performed using SPSS version 25.0. Descriptive analyses were conducted to assess means, standard deviations, distributions, linearity, and potential outliers. Primary analyses included simple mediation analyses using PROCESS, model 4 (Hayes, 2013). 5,000 bootstrap samples were requested along with a 95% confidence interval (CI). The total, direct, and indirect effects of the model were interpreted. Effect sizes were calculated by taking the ratio of indirect effect to total effect (direct + indirect effects) of x on y (Hayes, 2013), referred to as P_M_ (Preacher and Kelley, 2011). Please note, however, the controversy in the literature regarding comparability of mediation effects [e.g., (Preacher and Kelley, 2011)]. The SES composite served as the predictor, tests of executive function (i.e., Trails B, Digit Span Backwards, and Stroop) served as the outcome variables, and white matter tracts (i.e., ALIC, EC, SLF, and cingulum; Figure 1) served as independent mediators. Separate analyses were run for each test of executive function across each tract, yielding 12 models. For a theoretical model see Figure 2. Age was used as a covariate for all analyses.

A series of subsequent exploratory analyses were conducted on the models that were found to be significant in the primary analyses: (1) race and sex were included as additional covariates; (2) individual SES indicators (continuous education and poverty status) were used in place of the SES composite; (3) Trails A and Stroop Words were used as predictors in place of their executive function counterparts; (4) moderated mediations were run on the primary models to see if the relation between SES and white matter integrity varied by age. Continuous age was transformed into a binary variable (old, young) using the median. PROCESS model 7 was used; and (5) hypertension, diabetes, and BMI were added as additional covariates.

## Results

### Description of sample and study characteristics

Table 1 provides a description of the sample and study characteristics. Because of the large age range (33–72), we examined age distributions across race and SES groups. *M*(SD) for the low SES group = 50.30 (8.63) and the high SES group = 54.16 (9.53), which was significantly different (*t* = 2.99, p < 0.01). *M*(SD) for African Americans = 50.92 (10.17) and Whites = 53.45 (9.53), which was not significantly different (*t* = 1.92, p = 0.056).

**TABLE 1:** Study characteristics.

	Mean	SD	Percent	Range
Age (years)	52.36	9.30		33–72
% Male			44.8	
% White			56.7	
Education (years)	12.35	2.73		2–20
% High education			73.6	
% Above poverty			67.2	
% High SES			53.2	
Anterior limb of the internal capsule (FA)	0.39	0.02		0.34–0.46
External capsule (FA)	0.28	0.02		0.22–0.33
Superior longitudinal fasciculus (FA)	0.32	0.02		0.27–0.38
Cingulum bundle (FA)	0.21	0.02		0.16–0.25
Body mass index (kg/m^2^)	29.87	6.62		15.40–49.20
% Hypertension			47.3	
% Diabetes			15.9	
**Scores on neuropsychological tests**				
Trails A (time in seconds)	33.22	12.62		13–99
Trails B (time in seconds)	141.84	149.90		24–600
Digit span backwards (total score)	5.78	2.27		1–13
Stroop words (total score)	79.10	11.01		32–95
Stroop inhibition (total score)	31.69	10.09		8–62

SES, socioeconomic status; FA, fractional anisotropy; SD, standard deviation.

Analysis-specific samples varied based on complete performance for each cognitive measure: *n* = 198 for models with Trails B; *n* = 174 for models with Digit Span Backwards; n = 183 for models with Stroop. At *p* < 0.05, SES was associated with executive function and white matter integrity, Trails B was associated with the ALIC, EC, and SLF, and Stroop was associated with the ALIC and EC. There were no significant relations between Stroop and the SLF, Digit Span Backwards and any of the tracts, or the cingulum and any of the cognitive variables (Table 2).

**TABLE 2:** Matrix for correlation coefficients (Pearson’s r and r_pb_) for all model variables.

Variables	(1)	(2)	(3)	(4)	(5)	(6)	(7)	(8)	(9)	(10)	(11)	(12)	(13)	(14)
(1) SES	–	−0.21**	−0.18*	−0.15*	−0.22**	−0.16*	0.17*	−0.19*	−0.21**	−0.16*	0.13	–0.07	–0.13	–0.08
(2) Age		–	−0.15*	−0.18*	–0.05	–0.10	0.25**	–0.08	−0.20**	0.02	–0.14	0.02	0.21**	0.03
(3) ALIC			–	0.67***	0.51***	0.57***	−0.24**	–0.03	0.16*	0.12	–0.10	–0.02	−0.19**	−0.17*
(4) EC				–	0.60***	0.59***	−0.22**	0.01	0.21**	0.08	−0.16*	–0.05	−0.16*	–0.03
(5) SLF					–	0.56***	−0.16*	0.05	0.12	0.04	−0.19**	–0.08	–0.09	–0.04
(6) Cingulum						–	–0.11	–0.03	0.07	0.08	0.03	–0.01	0.00	–0.04
(7) Trails B							–	−0.43***	−0.49***	0.03	0.25**	–0.08	0.13	0.02
(8) DSB								–	0.45***	0.03	−0.19*	–0.09	–0.03	0.03
(9) Stroop									–	0.05	−0.15*	–0.09	–0.03	–0.06
(10) Sex										–	–0.04	–0.25	0.11	0.07
(11) Race											–	0.01	0.08	0.03
(12) BMI												–	0.21**	0.15*
(13) HTN													–	0.22**
(14) Diabetes														–

Pearson’s point-biserial correlations were used for associations between continuous and dichotomous variables. SES, socioeconomic status; ALIC, anterior limb of the internal capsule; EC, external capsule; SLF, superior longitudinal fasciculus; DSB, Digit Span Backwards; BMI, body mass index; HTN, hypertension. For SES, 0 = high SES and 1 = low SES; for sex, 0 = women and 1 = men; for race, 0 = White and 1 = African American; for hypertension 0 = no, 1 = yes; for diabetes 0 = no, 1 = yes. *p < 0.05; **p < 0.01; ***p < 0.001.

### Main analyses

**FIGURE 3:**
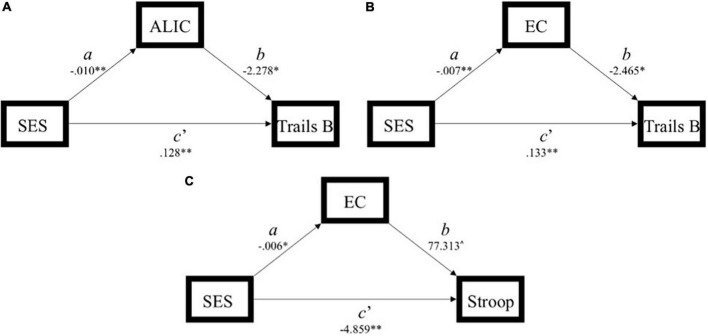
Simplified path model demonstration of models with a significant indirect effect (*ab*) of socioeconomic status (SES) on executive function through white matter integrity. **(A)** Model 1: significant indirect effect of SES on Trails B through the anterior limb of the internal capsule. **(B)** Model 4: significant indirect effect of SES on Trails B through the external capsule. **(C)** Model 6: significant indirect effect of SES on Stroop through the external capsule. SES, socioeconomic status; ALIC, anterior limb of the internal capsule; EC, external capsule.

With the exception of Trails A and B, there were no violations of assumptions of normality and linearity. Trails A and B were log transformed because they had a non-normal distribution. All reported coefficients were standardized unless otherwise noted. For brevity, only significant models are elaborated on here. Complete results are outlined in Table 3, and significant models are demonstrated in Figure 3.

**TABLE 3:** Summary of mediation models–main hypotheses.

	Path	*B*	*SE*	*t*	95% CI
Model 1					
	SES→(*c*) Trails B	0.475	0.044	3.435	0.064, 0.237***
	SES→(*a*) ALIC	–0.439	0.003	–3.097	−0.016, −0.004**
	ALIC→(*b*) Trails B	–0.164	0.956	–2.383	−4.162, −0.393*
	SES→(*c’*) Trails B	0.403	0.044	2.878	0.040, 0.215**
	Indirect effect (*ab*)	0.072	0.037*^b^*		0.018, 0.167*^a^*
Model 2					
	SES→(*c*) DSB	–0.415	0.344	–2.738	−1.622, −0.263**
	SES→(*a*) ALIC	–0.437	0.004	–2.911	−0.017, −0.003**
	ALIC→(*b*) DSB	–0.087	7.472	–1.124	−23.146, 6.354
	SES→(*c’*) DSB	–0.453	0.352	2.919	−1.725, −0.333**
	Indirect effect (*ab*)	0.038	0.039*^b^*		−0.022, 0.138
Model 3					
	SES→(*c*) Stroop	–0.528	1.456	–3.657	−8.197, −2.451***
	SES→(*a*) ALIC	–0.427	0.003	–2.887	−0.016, −0.003**
	ALIC→(*b*) Stroop	0.085	32.072	1.164	−25.954, 100.623
	SES→(*c’*) Stroop	–0.492	1.488	–3.334	−7.896, −2.024**
	Indirect effect (*ab*)	–0.036	0.034*^b^*		−0.117, 0.019
Model 4					
	SES→(*c*) Trails B	0.475	0.044	3.435	0.064, 0.237***
	SES→(*a*) EC	–0.384	0.003	–2.706	−0.012, −0.002**
	EC→(*b*) Trails B	–0.146	1.170	–2.108	−4.772, −0.159*
	SES→(*c’*) Trails B	0.419	0.044	3.001	0.046, 0.220**
	Indirect effect (*ab*)	0.056	0.033*^b^*		0.007, 0.145*^a^*
Model 5					
	SES→(*c*) DSB	–0.415	0.344	–2.738	−1.622, −0.263**
	SES→(*a*) EC	–0.368	0.003	–2.455	−0.013, −0.001*
	EC→(*b*) DSB	–0.045	9.262	–0.587	−23.717, 12.852
	SES→(*c’*) DSB	–0.432	0.351	–2.794	−1.674, −0.288**
	Indirect effect (*ab*)	0.017	0.038*^b^*		−0.042, 0.115
Model 6					
	SES→(*c*) Stroop	–0.528	1.456	–3.657	−8.197, −2.451***
	SES→(*a*) EC	–0.328	0.003	–2.216	−0.011, −0.001*
	EC→(*b*) Stroop	0.141	39.655	1.950	-0.939, 155.564^
	SES→(*c’*) Stroop	–0.482	1.464	–3.318	−7.749, −1.970**
	Indirect effect (*ab*)	–0.046	0.323*^b^*		−0.137,−0.001*^a^*
Model 7					
	SES→(*c*) Trails B	0.475	0.044	3.435	0.064, 0.237***
	SES→(*a*) SLF	–0.484	0.003	–3.396	−0.016, −0.004***
	SLF→(*b*) Trails B	–0.104	1.070	–1.499	−3.715, 0.506
	SES→(*c’*) Trails B	0.425	0.045	2.994	0.050, 0.223**
	Indirect effect (*ab*)	0.050	0.042*^b^*		−0.021, 0.147
Model 8					
	SES→(*c*) DSB	–0.415	0.344	–2.738	−1.622, −0.263**
	SES→(*a*) SLF	–0.443	0.003	–2.931	−0.016, −0.003**
	SLF→(*b*) DSB	–0.003	8.308	–0.042	−16.750, 16.051
	SES→(*c’*) DSB	–0.416	0.354	–2.674	−1.645, −0.248**
	Indirect effect (*ab*)	0.001	0.042*^b^*		−0.073, 0.094
Model 9					
	SES→(*c*) Stroop	–0.528	1.456	–3.657	−8.197, −2.451***
	SES→(*a*) SLF	–0.432	0.003	–2.900	−0.015, −0.003**
	SLF→(*b*) Stroop	0.061	35.205	0.841	−39.852, 99.090
	SES→(*c’*) Stroop	–0.501	1.491	–3.394	−8.001, −2.118***
	Indirect effect (*ab*)	–0.026	0.038*^b^*		−0.118, 0.039
Model 10					
	SES→(*c*) Trails B	0.475	0.044	3.435	0.064, 0.237***
	SES→(*a*) Cingulum	–0.382	0.003	–2.658	−0.012, −0.002**
	CB→(*b*) Trails B	–0.042	1.242	–0.604	−3.199, 1.699
	SES→(*c’*) Trails B	0.459	0.045	3.256	0.057, 0.234***
	Indirect effect (*ab*)	0.016	0.030*^b^*		−0.032, 0.092
Model 11					
	SES→(*c*) DSB	–0.415	0.344	–2.738	−1.622, −0.263**
	SES→(*a*) Cingulum	–0.377	0.003	–2.490	−0.012, −0.001*
	CB→(*b*) DSB	–0.082	9.839	–1.074	−29.987, 8.859
	SES→(*c’*) DSB	–0.446	0.350	–2.892	−1.705, −0.322**
	Indirect effect (*ab*)	0.031	0.038*^b^*		−0.021, 0.141
Model 12					
	SES→(*c*) Stroop	–0.528	1.456	–3.657	−8.197, −2.451***
	SES→(*a*) Cingulum	–0.391	0.003	–2.623	−0.012, −0.002**
	CB→(*b*) Stroop	0.001	41.372	0.008	−81.315, 81.966
	SES→(*c’*) Stroop	–0.527	1.488	–3.578	−8.258, −2.387***
	Indirect effect (*ab*)	–0.000	0.035*^b^*		−0.072, 0.071

*c* is the total effect of SES on the relevant outcome variable; the path coefficients (*a, b, c’*) estimate the strength of hypothesized causal associations; *c’* is the direct effect of SES on the relevant outcome variable; *ab* estimates the strength of the indirect effect of SES on the relevant outcome variable through the mediating tract. There are no *t*-or *p*-values produced for the indirect effects. All models are adjusted for age. All coefficients are standardized; indirect effects are partially standardized because the predictor variable is dichotomous. SES, socioeconomic status; ALIC, anterior limb of the internal capsule; EC, external capsule; SLF, superior longitudinal fasciculus; DSB, Digit Span Backwards.

^*a*^Confidence intervals did not cross zero; ^*b*^Bootstrapping based standard errors.

*p < 0.05; **p < 0.01; ***p < 0.001; ^*p* = 0.05.

#### Anterior limb of the interior capsule

In Model 1, the partially standardized indirect effect (*ab*) of SES on Trails B *via* the ALIC, controlling for age, was 0.072 (*P*_M_ = 0.152; CI: 0.018; 0.167). As the bootstrapped CI did not include zero, the indirect effect was statistically significant. The direct path from SES to Trails B (*c’*) was also significant, and therefore, the effect of SES on Trails B was partially mediated by the ALIC. These findings held using Trail B’s traditional 300-s cutoff.

The ALIC did not mediate the SES-Digit Span Backwards or the SES-Stroop relations.

#### External capsule

In Model 4, the partially standardized indirect effect (*ab*) of SES on Trails B *via* the EC, controlling for age, was 0.056 (*P*_M_ = 0.118; CI: 0.007; 0.145). As the bootstrapped CI did not include zero, the indirect effect of SES on Trails B through the EC was statistically significant. The direct path from SES to Trails B (*c’*) was also significant, and therefore, the effect of SES on Trails B was partially mediated by the EC. These findings held using Trail B’s traditional 300-s cutoff.

In Model 6, the partially standardized indirect effect (*ab*) of SES on Stroop *via* the EC, controlling for age, was −0.046 (*P*_M_ = 0.087; CI: –0.137; –0.001). As the bootstrapped CI did not include zero, the indirect effect of SES on Stroop through the EC was statistically significant. The direct path from SES to Stroop (*c’*) was also significant, and therefore, the effect of SES on Stroop was partially mediated by the integrity of the EC.

The EC did not mediate the SES-Digit Span Backwards relation.

#### Cingulum

The Cingulum did not mediate any of the SES-cognition relations.

#### Superior longitudinal fasciculus

The SLF did not mediate any of the SES-cognition relations.

### Exploratory analyses

The same model characteristics were employed for all exploratory analyses unless otherwise noted. Effect sizes for all models are reported in Table 4.

**TABLE 4:** Effect size comparisons between original and adjusted models.

	Original	Model A	Model B	Model C	Model D	Model F	Model G	Model H
Model 1	0.1521	0.1289	0.0943	0.1618	0.105	0.139	0.161	0.157
Model 4	0.1179	0.0826	0.0620	0.1254	0.061	0.108	0.119	0.121
Model 6	0.0874	0.0725	0.0821	0.0804	0.131	0.097	0.084	0.083

Effect size is the ratio of indirect effect to total effect (direct + indirect effects) of x on y. For example, in Model 1’s original model, 15.21% of the significant total effect of SES on Trails B is accounted for by integrity of the ALIC. Effect sizes could not be calculated for Models E because there are no total effects provided in moderated mediation models.

#### Covarying race and sex

Sensitivity analyses were performed to test the influence of race and sex on the significant mediations. Sex did not change the statistical significance of any of the models. Race did not change the statistical significance of Model 1a (*P*_M_ = 0.129; CI: 0.008, 0.145), but rendered the mediations in Models 4a (*P*_M_ = 0.083; CI: −0.003, 0.115) and 6a (*P*_M_ = 0.073; CI: −0.120, 0.007) non-significant. Complete results are reported in Supplementary Table 1. To further understand the results of Models 4a and 6a, *post hoc* analyses were conducted substituting race for SES as the independent variable. The models were then further adjusted for SES.

##### Race→EC→Trails B with further adjustment of socioeconomic status

The partially standardized indirect effect (*ab*) of race on Trails B *via* the EC, controlling for age was 0.048 (CI: 0.002; 0.140). As the bootstrapped CI did not include zero, the indirect effect of race on Trails B through the EC was statistically significant. The direct path from race to Trails B (*c’*) was also significant, and therefore, the effect of race on Trails B was partially mediated by the EC. After SES was added, the indirect effect decreased to 0.032 (CI: −0.005; 0.117) and was no longer statistically significant.

##### Race→EC→Stroop with further adjustment of socioeconomic status

The partially standardized indirect effect (*ab*) of race on Stroop *via* the EC, controlling for age, was –0.049 (CI: −0.150; −0.002). As the bootstrapped CI did not include zero, the indirect effect of race on Stroop through the EC was statistically significant. The direct path from race to Stroop (*c’*) was also significant, and therefore, the effect of race on Stroop was partially mediated by the EC. After SES was added, the indirect effect decreased to –0.035 (CI: −0.139; 0.008) and was no longer statistically significant.

#### Individual socioeconomic status indicators as the predictor variable

Socioeconomic status (SES) was replaced with individual SES indicators (continuous education, poverty status). See Supplementary Table 2. For continuous education, the results were like the primary models. The indirect effects of Models 1b (*P*_M_ = 0.094; CI: −0.076, −0.005), 4b (*P*_M_ = 0.062; CI: −0.056, −0.001), and 6b (*P*_M_ = 0.082; CI: 0.001, 0.067) were significant. For poverty status, the indirect effects of Models 1c (*P*_M_ = 0.162; CI: 0.007, 0.153) and 4c (*P*_M_ = 0.125; CI: 0.003, 0.136) were significant, but model 6c (*P*_M_ = 0.080; CI: −0.126, 0.007) was not.

#### Processing speed as the outcome variable

Trails B and Stroop Inhibition were replaced with their processing speed counterparts, Trails A and Stroop Words, respectively. See Supplementary Table 3. The Trails A→SES→ALIC model (Model 1d) was significant (*P*_M_ = 0.105; CI: 0.014, 0.157), however the Trails A→SES→EC (4d; *P*_M_ = 0.061; CI: −0.009, 0.136) and the Stroop Words→SES→EC (6d; *P*_M_ = 0.131; CI: −0.149, 0.035) models were not.

#### Socioeconomic status by age moderated mediations

To see if age moderated the SES by white matter path in the primary models, moderated mediations were conducted. See Supplementary Table 4 for results. None of the moderated mediations were significant.

#### Covarying cardiovascular variables

Please see Supplementary Table 5. When covarying the cardiovascular variables, indirect effects for the models with Trails B as the outcome variable, 1f (*P*_M_ = 0.139; CI: 011, 0.158), 4f (*P*_M_ = 0.108; CI: 0.003, 0.134), 1g (*P*_M_ = 0.161; CI: 0.017, 0.173), 4g (*P*_M_ = 0.119; CI: 0.007, 0.142), 1h (*P*_M_ = 0.157; CI: 0.015, 0.165), and 4h (*P*_M_ = 0.121; CI: 0.008, 0.147) remained significant. Indirect effects for the models with Stroop, 6f (*P*_M_ = 0.097; CI: −0.150, 0.002), 6g (*P*_M_ = 0.084; CI: −0.138, 0.004), and 6h (*P*_M_ = 0.083; CI: −0.133, 0.003), lost significance.

## Discussion

This study examined whether the relation between SES and executive function was mediated by white matter integrity in a socioeconomically diverse sample of community-dwelling adults. Low SES was associated with poorer executive function and white matter integrity. Lower Trails B performance was associated with poorer integrity of the ALIC, EC, and SLF, and lower Stroop performance was associated with poorer integrity of the ALIC and EC. There were no significant relations between Stroop and the SLF, Digit Span Backwards and any of the tracts, or the cingulum and any of the cognitive variables. Out of the twelve primary models, three demonstrated significant mediations: the ALIC partially mediated the relation between SES and Trails B, and the EC partially mediated relations between SES and Trails B, and between SES and Stroop.

### Socioeconomic status and executive function

Results of the current study indicate that lower SES was associated with poorer performance on several tests of executive function assessing set-shifting, response inhibition, and working memory, independent of age. These findings add to the literature showing that higher SES is related to better executive function. The disadvantage associated with poorer socioeconomic conditions, particularly during childhood, has a profound impact on executive function-sensitive regions and circuits, perhaps given their relatively longer period of development (Giedd, 2004; Gogtay et al., 2004; Sowell et al., 2004; Blakemore and Choudhury, 2006). The mechanistic pathways by which these cognitive disparities occur are likely multifactorial, lifelong, and bidirectional (Farah, 2017). Among many contributors, poor nutrition, reduced cognitive stimulation, increased toxin exposure, lower access to health care, higher incidence of physical disease, and higher rates of stressors are thought to underlie SES-related cognitive differences. These factors are linked to physiological changes in the brain and other organ systems known to contribute to cognitive difficulties. As discussed below, perhaps variability in white matter integrity is one of the biological changes that accounts for these cognitive disparities.

### Socioeconomic status and white matter integrity

The associations of SES with white matter integrity in the ALIC, SLF, EC, and cingulum are consistent with literature demonstrating SES-white matter relations in these (Gianaros et al., 2013; Ursache and Noble, 2016; Dufford and Kim, 2017; Takeuchi et al., 2018) and other fiber tracts (Teipel et al., 2009; Chiang et al., 2011; Johnson et al., 2013; Dufford and Kim, 2017), as well as in the brain more broadly (Jednorog et al., 2012; Shaked et al., 2019a). These results contribute to the nascent literature demonstrating that poorer socioeconomic conditions are related to lesser white matter integrity.

While the mechanistic pathways for socioeconomic disparities in white matter microstructure are largely unexamined, the literature examining SES-related influences on other neuroanatomical outcomes may shed light on potential pathways. Among many biopsychosocial factors, mechanistic influences may include stress, cardiovascular risk factors and other medical conditions, adverse environmental exposures (e.g., toxins), cognitive stimulation, and access to resources. One of the only studies examining these pathways found that cigarette smoking, larger waist circumference, and higher C-reactive protein partly accounted for the relation between SES and white matter integrity in adults (Gianaros et al., 2013). The authors argued that modifiable risk factors like nutrition, exercise, and tobacco use might explain disparities in white matter integrity. These findings suggest a possible SES to white matter pathway, whereby various disadvantages associated with lower SES relate to white matter degradation. It is likely that the SES-white matter associations are bidirectional, but longitudinal studies are needed to better identify directionality.

### White matter integrity and executive function

Connections between Trails B and integrity of the ALIC, EC, and SLF, as well as Stroop and integrity of the ALIC and EC are consistent with the well-established disconnection hypothesis, which suggests that deterioration of white matter tracts results in impaired information transfer across brain regions resulting in cognitive difficulties (O’Sullivan et al., 2001). Executive functions are particularly vulnerable to dysconnectivity (Correia et al., 2008; Zahr et al., 2009; Voineskos et al., 2012), perhaps because they rely on lower-order abilities *via* communication with multiple regions across the entire brain. It may be that certain subdomains of executive function are more susceptible to dysconnectivity in the tracts examined here. Consistent with prior literature (Jacobs et al., 2013; Muir et al., 2015), set-shifting and response inhibition may be sensitive to integrity of the ALIC and EC, although few studies have examined the EC in this context.

Importantly, the cingulum was not associated with the examined tests of executive function, and the SLF was only associated with Trails B. The null findings may be due to the large length and width of the cingulum and SLF. These tracts are considered multicomponent bundles with distinct functional and anatomical subdomains (Catani and Thiebaut de Schotten, 2012). Perhaps significant cognition-white matter relations are specific to portions of the tracts that were not isolated in this study due to image processing constraints. For instance, relative to its other branches, the SLF II may be particularly important for executive function, including manipulation of visuospatial information (Catani and Thiebaut de Schotten, 2012). It is therefore not surprising that out of the three tests examined, Trails B was related to the SLF, as one of the cognitive abilities required for Trails B is visuospatial manipulation. SLF-Trails B associations are consistent with prior work (Jacobs et al., 2013; Muir et al., 2015). Given that the SLF II supports other executive function subdomains like attention, perhaps examination of the SLF II in isolation would reveal relations to Stroop and Digit Span Backwards. The cingulum also has multiple components. Given its anatomical location, the anterior portion seems to be most important for executive function abilities, including cognitive control (Catani and Thiebaut de Schotten, 2012). Given the heterogeneity of these tracts, it is plausible that lack of specific segmentation prevents the discovery of potentially important relations between the cognitive tests examined here and the tracts’ subcomponents.

### White matter’s mediating role

#### Anterior limb of the internal capsule’s mediating role on the socioeconomic status-Trails B relation

The ALIC (Figure 1, Panel A) contains the anterior thalamic radiation, which links the mediodorsal and anterior thalamic nuclei with the prefrontal cortex and the cingulate gyrus (Parent, 1996; Zhou et al., 2003). Frontothalamic connectivity may therefore be relevant to Trails B performance. This tract involves brain regions thought to be important for executive function, including the prefrontal cortex, anterior cingulate cortex, and mediodorsal thalamic nucleus (the major thalamic relay to the frontal cortex; Blumenfeld, 2010). Thus, dysconnectivity of these brain regions may result in poorer Trails B performance. Interestingly, the ALIC is the tract connecting the thalamus with the frontal eye field, which is involved in voluntary eye movements and gaze fixation necessary for attention (Schoenberg and Scott, 2011). Given the visuospatial and attentional components of Trails B, perhaps it also relies on efficient connectivity between the frontal eye field and other brain areas. Accordingly, the Trails B-ALIC relation helps explain this significant mediation. Results are consistent with studies finding relations between Trails B and ALIC (Jacobs et al., 2013), SES and ALIC (Gianaros et al., 2013; Dufford and Kim, 2017), and Trails B and SES (Lipina et al., 2001; Turrell et al., 2002). Ultimately, lower levels of cognitive flexibility found in individuals from lower SES homes may be partly explained by poorer integrity of the ALIC.

After adjusting for race and sex, this mediation retained its significance, which is perhaps not surprising given no significant sex differences in SES, ALIC or Trails B performance. There was a significant main effect of race on Trails B performance, suggesting that the ALIC accounts for a significant amount of variance over and above the contribution of race on Trails B performance. There was no variability in the ALIC across racial groups, suggesting that the ALIC disparity may be unique to socioeconomic differences.

#### External capsule’s mediating role on the socioeconomic status-Trails B and socioeconomic status-Stroop relations

The EC connects the basal forebrain with the rest of the brain (Figure 1, Panel B). The basal forebrain is central to the production of acetycholine, which is then distributed throughout the brain, in part by the EC. Acetycholine and the cholinergic system more generally are important for executive functions (Logue and Gould, 2014) and disruption of cholinergic signaling has been shown to impact cognitive flexibility in rats (Chen et al., 2004; McLean et al., 2012). Given that acetycholine acts on nicotinic receptors, at least two studies have shown that disruption in acetycholine release impacts response inhibition in smokers, including on the Stroop (Wignall and de Wit, 2011; Rhodes et al., 2012). Moreover, findings are consistent with studies showing EC’s direct relation with cognition (O’Brien et al., 2002; Mabbott et al., 2006; Kraus et al., 2007), as well as with income (Gianaros et al., 2013) and early life neglect (Bick et al., 2015). The EC mediated the relation between SES and performance on two executive function measures, suggesting that it is perhaps an essential biological underpinning of the SES-cognition relation.

Further adjustments revealed that these mediations lost their significance after adjustment for race but not sex. Sex was unrelated to any of the variables, while race was related to the EC, Stroop, and Trails B. Table 5 compares the mediation paths in the original model to those in the race and sex-adjusted exploratory models, showing that in the models with EC as a mediator, the EC to cognition paths became non-significant after accounting for race. Thus, the EC was no longer associated with Trails B and Stroop. To better understand this, the models were reversed as to designate race as the independent variable, and SES as the further adjustment variable. Interestingly, these flipped models demonstrated the same pattern of findings: the EC significantly mediated the relations between race and Trails B and race and Stroop, but when further adjusting the model by SES the mediations lost their significance. The consistency of findings across models suggests that race and SES have a substantial amount of overlapping influence on these mediational pathways.

**TABLE 5:** Path comparisons between original and adjusted models.

		Original model		Adjusted model	
			
		*B*	*sr* ^2^	*B*	*sr* ^2^
Model 1					
	SES→ALIC	–0.220	0.047**	–0.195	0.035**
	ALIC→Trails B	–0.164	0.025*	–0.145	0.019*
	SES→Trails B	0.201	0.037**	0.193	0.033**
Model 4					
	SES→EC	–0.197	0.037**	–0.172	0.027*
	EC→Trails B	–0.146	0.020*	–0.107	0.010
	SES→Trails B	0.209	0.040**	0.203	0.037**
Model 6					
	SES→EC	–0.197	0.037**	–0.172	0.027*
	EC→Stroop	0.141	0.018^	0.117	0.013
	SES→Stroop	–0.240	0.054**	–0.231	0.045**

Data reflect standardized regression coefficients (B) and semipartial correlations squared (*sr*^2^). SES, socioeconomic status; ALIC, anterior limb of the internal capsule; EC, external capsule. *p < 0.05, **p < 0.01, ***p < 0.001, ^*p* = 0.05.

Interestingly, correlational results (Table 2) showed that while all the white matter tracts were related to SES, only two of the four tracts (EC, SLF) were related to race. Previous findings by our group (Shaked et al., 2019a) demonstrated SES disparities in white matter integrity across the four primary brain lobes (parietal, temporal, occipital, frontal), but no racial disparities across these regions. It was noted that while social risk factors specifically linked to race, such as racial discrimination, adversely impact global and regional brain and white matter volumes, they may be less related to white matter integrity. This hypothesis, however, was reported to be highly speculative. The correlations found in this study demonstrating more consistent associations with SES, relative to race, once again suggest that perhaps relative to the biopsychosocial risk associated with race, those related to SES exert greater influence on white matter integrity. This is nonetheless, once again, highly speculative, and an important question to be examined in future research.

While race did not differ significantly across SES groups in the present sample (*p* = 0.072), it is possible that this study is not able to fully disentangle the independent effects of race and SES. Furthermore, researchers have discussed the great challenge of fully distinguishing race and SES given how confounded they are (Manly, 2006; Glymour and Manly, 2008; Williams et al., 2010). For example, even if there is no difference in years of education received across racial groups, the quality of education is likely discordant due to a history of segregation and unequal allocation of funds in White versus African American schools (Manly, 2006). Also, there are substantial SES-and race-related health disparities, and many of the mechanistic pathways by which these inequities occur seem common across race and SES (Williams et al., 2010). In addition to unique race-related stressors like racism, African Americans across the socioeconomic spectrum undergo environmental challenges similar to those with low SES. For example, relative to Whites, African Americans are more likely to have higher rates of stress, fewer high-quality educational opportunities, and less access to healthy foods (Glymour and Manly, 2008; Williams et al., 2010). This disadvantage is known to adversely impact health and cognitive outcomes in both African Americans and individuals living in low SES (Adler et al., 1994; Williams et al., 2010). Given that SES and race share a similar pattern of influence on neurocognitive outcomes, it is perhaps not surprising that the mediation findings appear similar across the models, and that controlling for one another renders the findings non-significant.

### Influence of individual socioeconomic status indicators

Exploratory analyses revealed that when education and poverty status were used as predictors in place of the SES composite, results were similar. The one exception was that the external capsule did not mediate the poverty status-Stroop relation. Different socioeconomic influences play varying roles on brain plasticity (Farah, 2017). Poverty status is a proxy for one’s economic status, such as access to medical, health, and other resources, job availability and security, and exposure to environmental toxins. While educational attainment also captures one’s economic condition, it represents school-based factors like literacy and cognitive stimulation. We selected the SES composite as our primary SES indicator given that prior work has shown that it is a strong determinant of health disparities (Adler et al., 1994; Waldstein et al., 2017). These exploratory results further our understanding by demonstrating that both poverty status and level of education are important in explaining disparities in white matter integrity and cognition.

### Processing speed versus executive function

To better understand if the significant mediations were unique to executive function, we replaced the executive function variables with their processing speed counterparts (i.e., Trails A for Trails B; Stroop Words for Stroop Inhibition). ALIC significantly mediated the SES-Trails A relation, but the EC did not mediate the SES-Trails A nor the SES-Stroop Words relations. It is plausible that processing speed is also impacted by SES and white matter disparities. Indeed, prior literature has shown that among many white matter tracts, ALIC integrity is associated with processing speed (Chopra et al., 2018). As the EC mediations were not significant, perhaps executive function is uniquely important here, however further research is needed to understand the differential impact of SES and white matter integrity across cognitive domains. Ultimately, as executive function and processing speed are often highly correlated (e.g., Albinet et al., 2012) and because white matter integrity is associated with both executive function and processing speed (Santiago et al., 2015), it is likely that both domains are impacted by the disparities addressed in this paper.

### Socioeconomic status by age moderated mediations

Given the large age range in our dataset, we ran moderated mediations to see if the relation between SES on white matter integrity was dependent on age. None of the moderated mediations were significant, suggesting that in our sample, the effect of SES on white matter integrity does not vary by age.

### Cardiovascular risk factors

Sensitivity analyses revealed that adjustment of cardiovascular risk factors consistently eliminated the indirect effects of Model 6 (SES→EC→Stroop), but not Models 1 (SES→ALIC→Trails B) or 4 (SES→ALIC→Stroop). Bivariate correlations did not suggest stronger relations between the cardiovascular risk factors with the EC versus the ALIC, or with Stroop versus Trails B (Table 1). Therefore, the most parsimonious explanation for the weakening of the indirect effect in Model 6 is statistical power, particularly as in the primary analyses this model had a smaller indirect effect relative to the other models (Table 4).

Although the indirect effects of Models 1 and 4 remained significant after adjusting for BMI, diabetes, and hypertension, cardiovascular and other biomedical health factors likely play an important role in the SES-white matter-cognition associations. Examining these relations using additional cardiovascular biomarkers and risk scores (e.g., Framingham Risk Score) and latent variable modeling (e.g., structural equation modeling), with a larger sample size, would better our understanding of these complex pathways.

### Null findings

The lack of mediations with Digit Span Backwards may be due to the limited variability in test performance (Table 1) that makes it more difficult to identify potentially important relations. Also, the test’s distribution of scores in our sample is generally lower than studies using more sociodemographically homogenous samples (Kemtes and Allen, 2008), suggesting that prior results may not be as relevant to our diverse sample. Moreover, white matter tracts not examined here (e.g., fornix, uncinate fasciculus, medial temporo-frontal pathway) may be more supportive of this test’s function (Zahr et al., 2009; Charlton et al., 2010). As noted above, the null findings related to the Cingulum and SLF may be due to their size and multicomponent nature.

### Strengths and limitations

There are various strengths to this study. First, the unique methodological approach to the HANDLS investigation allowed us to ask important health-related questions in a sociodemographically diverse sample. Also, for an imaging study this is a relatively large sample. Moreover, this study examined four distinct white matter tracts, and three measures of executive function, which allowed for a more nuanced investigation on the specific tracts and executive function components that are most relevant in this context. Additionally, the study sample was comprised of adults, which compared to children, is a relatively understudied population in the SES and brain literature. Finally, this is the first study to employ the mediation models presented here in adults, and the second study to use this model in the broader literature (Ursache and Noble, 2016).

There are several limitations to the current study. First, while our SES composite captures both poverty status and education, it does not measure SES continuously. HANDLS does not have a full spectrum of SES based on specific annual income, in part due to participants having difficulty reporting their annual income. Also, continuous income does not account for household size. Poverty status, a specific income level adjusted for household size, was therefore used as HANDLS’ primary stratification level. Moreover, while our imaging subsample was representative of the larger HANDLS sample with regards to sex, the imaging subsample was significantly more likely to include younger, White participants, who live above the poverty line. Conclusions regarding race, SES, and age effects should therefore be generalized with caution to the overall HANDLS sample. Also, this study only examined *a priori* a select few white matter tracts and cognitive measures. Moreover, HANDLS’ dementia exclusion criteria was limited to the Mini-Mental State Examination, a screening measure which cannot reliably diagnose or rule-out dementia, particularly in samples with minoritized sociodemographic groups. Additionally, our group contrasts were not corrected for multiple comparisons due to concerns regarding Type 2 error in this novel and largely exploratory study. Thus, the chance of Type 1 error remains a concern. Finally, this study was cross-sectional and therefore did not examine age-related changes in white matter integrity and cognition.

## Conclusion

Marginalized individuals who are continuously exposed to adverse biopsychosocial factors (e.g., acute and prolonged environmental stressors, higher incidence of disease, discrimination, less access to resources like healthy food and medical care, greater toxin exposure) are at a disadvantage with regards to achieving and maintaining optimal levels of cognitive outcomes. Environmental inequities put individuals from low SES homes at greater risk for developing physiological changes, including poorer brain outcomes, which tend to adversely impact cognitive development and perpetuate cognitive decline. This study demonstrates one of the complex pathways by which marginalized individuals experience cognitive disparities and advances our understanding of the complex interrelations between SES, brain, and cognition. These results may encourage future work that examines individuals longitudinally to determine neurodevelopmental and biopsychosocial influences on these relations across the lifespan. Clarifying mechanistic pathways by which these disparities occur may encourage researchers, clinicians, and policy makers to prioritize prevention and intervention efforts that facilitate brain and cognitive health among those at greatest risk.

## Data availability statement

The original contributions presented in the study are included in the article, further inquiries can be directed to the corresponding author.

## Ethics statement

The studies involving human participants were reviewed and approved by the Institutional Review Board (IRB) of the National Institutes of Health. The patients/participants provided their written informed consent to participate in this study.

## Author contributions

DS and SW: general conception. ME, AZ, and SW: parent study design. DS: data analysis and drafting the manuscript. All authors: data collection, preparation, interpretation, and final preparation of the manuscript.
