# Supplementary material for: White matter integrity as a mediator between socioeconomic status and executive function
Source: Front Hum Neurosci. 2022 Nov 18;16:1021857. doi: 10.3389/fnhum.2022.1021857 (PMC9716285; doi:10.3389/fnhum.2022.1021857)
Supplement: Supplementary file 1 [file Data_Sheet_1.pdf]

Supplementary Table 1. Mediation Models adjusted for Age, Race, and Sex

	Path	*B*	*SE*	*t*	95% CI
Model 1a					
	SES ➔ (*c*) Trails B	.443	.043	3.280	.056, .225**
	SES ➔ (*a*) ALIC	-.392	.003	-2.725	-.015, -.003**
	ALIC ➔ (*b*) Trails B	-.146	.928	-2.172	-3.848, -.186*
	SES ➔ (*c'*) Trails B	.386	.043	2.831	.037, .208**
	**Indirect Effect (*ab*)**	**.057**	**.033**[b]		**.008, .145**[a]
Model 4a					
	SES ➔ (*c*) Trails B	.443	.043	3.280	.056, .225**
	SES ➔ (*a*) EC	-.344	.003	-2.400	-.012, -.001*
	EC ➔ (*b*) Trails B	-.107	1.144	-1.580	-4.063, .448
	SES ➔ (*c'*) Trails B	406	.043	2.976	.043, .214**
	**Indirect Effect (*ab*)**	**.037**	**.029**[b]		**-.003, .115**
Model 6a					
	SES ➔ (*c*) Stroop	-.500	1.460	-3.455	-7.926, -2.163***
	SES ➔ (*a*) EC	-.310	.003	-2.080	-.011, -.000*
	EC ➔ (*b*) Stroop	.117	39.811	1.615	-14.262, 142.868
	SES ➔ (*c'*) Stroop	-.464	1.471	-3.180	-7.582, -1.775**
	**Indirect Effect (*ab*)**	**-.036**	**.031**[b]		**-.120, .007**

c is the total effect of SES on the relevant outcome variable; the path coefficients (*a*, *b, c'*) estimate the strength of hypothesized causal associations; *c'* is the direct effect of SES on the relevant outcome variable; *ab* estimates the strength of the indirect effect of SES on the relevant outcome variable through the mediating tract. There are no t- or p-values produced for the indirect effects. All models are adjusted for age, sex, and race. All coefficients are standardized; indirect effects are partially standardized because the predictor variable is dichotomous. SES = socioeconomic status; ALIC = anterior limb of the internal capsule; EC = external capsule.
[a]Confidence intervals did not cross zero; [b]Bootstrapping based standard errors. *$p < .05$; **$p < .01$; ***$p<.001$

Supplementary Table 2. Mediation Models with Individual SES Indicators

	Path	*B*	*SE*	*t*	95% CI
Continuous Education					
Model 1b					
	Education ➔ (*c*) Trails B	-.313	.008	-4.728	-.053, -.022***
	Education ➔ (*a*) ALIC	.194	.001	2.768	.001, .003**
	ALIC ➔ (*b*) Trails B	-.152	.929	-2.267	-3.938, -.274*
	Education ➔ (*c'*)Trails B	-.284	.008	-4.245	-.050, -.018***
	**Indirect Effect (*ab*)**	**-.029**	**.002**[b]		**-.076, -.005**[a]
Model 4b					
	Education ➔ (*c*) Trails B	-.313	.008	-4.728	-.053, -.022***
	Education ➔ (*a*) EC	.134	.001	1.900	.000, .002
	EC ➔ (*b*) Trails B	-.145	1.129	-2.178	-4.686, -.231*
	Education ➔ (*c'*)Trails B	-.294	.008	-4.436	-.050, -.019***

	**Indirect Effect (*ab*)**	**-.019**	**.014[b]**		**-.056, -.001[a]**
Model 6b					
	Education ➔ (*c*) Stroop	.255	.263	3.595	.427, -1.467***
	Education ➔ (*a*) EC	.144	.001	1.980	.000,.002*
	EC ➔ (*b*) Stroop	.145	39.566	2.015	1.659, 157.812*
	Education ➔ (*c'*) Stroop	.234	.264	3.293	.348, 1.390**
	**Indirect Effect (*ab*)**	**.021**	**.016[b]**		**.001, .067[a]**
Poverty Status					
Model 1c					
	Pov Stat ➔ (*c*) Trails B	.372	.047	2.498	-.053, -.022*
	Pov Stat ➔ (*a*) ALIC	-.324	.004	-2.119	-.014, .-.001*
	ALIC ➔ (*b*) Trails B	-.186	.953	-2.700	-4.451, -.693**
	Pov Stat ➔ (*c'*)Trails B	.312	.047	2.104	.006, .192*
	**Indirect Effect (*ab*)**	**.060**	**.035[b]**		**.007, .153[a]**
Model 4c					
	Pov Stat ➔ (*c*) Trails B	.372	.047	1.498	.025, .211*
	Pov Stat ➔ (*a*) EC	-.284	.003	-1.859	-.011, .000
	EC ➔ (*b*) Trails B	-.165	1.171	-2.838	-5.103, -.486*
	Pov Stat ➔ (*c'*)Trails B	.326	.047	2.190	.010, .196*
	**Indirect Effect (*ab*)**	**.047**	**.031[b]**		**.003, .136 [a]**
Model 6c					
	Pov Stat ➔ (*c*) Stroop	-.427	1.609	-2.676	-7.480, -1.130**
	Pov Stat ➔ (*a*) EC	-.211	.003	-1.302	-.010,.002
	EC ➔ (*b*) Stroop	.162	39.824	2.241	10.646, 167.814*
	Pov Stat ➔ (*c'*) Stroop	-.392	1.599	-2.476	-7.113, -.804*
	**Indirect Effect (*ab*)**	**-.036**	**.031[b]**		**-.126, .007**

c is the total effect of SES on the relevant outcome variable; the path coefficients (*a, b, c'*) estimate the strength of hypothesized causal associations; *c'* is the direct effect of SES on the relevant outcome variable; *ab* estimates the strength of the indirect effect of SES on the relevant outcome variable through the mediating tract. There are no t- or p-values produced for the indirect effects. All models are adjusted for age. All coefficients are standardized; indirect effects are completely standardized. ALIC = anterior limb of the internal capsule; EC = external capsule. [a]Confidence intervals did not cross zero; [b]Bootstrapping based standard errors. *$p < .05$; **$p < .01$; ***$p < .001$

Supplementary Table 3. Mediation Models with Processing Speed as the Outcome Variable

	Path	*B*	*SE*	*t*	95% CI
Model 1d					
	SES ➔ (*c*) Trails A	.656	.019	4.831	.055, .130***
	SES ➔ (*a*) ALIC	-.436	.003	-3.080	-.016, -.004**
	ALIC ➔ (*b*) Trails A	-.158	.418	-2.333	-1.798, -.151*
	SES ➔ (*c'*) Trails A	.588	.019	4.270	.044, .121***
	**Indirect Effect (*ab*)**	**.069**	**.036[b]**		**.014, .157[a]**
Model 4d					
	SES ➔ (*c*) Trails A	.656	.019	4.831	.055, .130***

	Path	B	SE	t	95% CI
	SES ➔ (*a*) EC	-.370	.003	-2.607	-.012, -.002**
	EC ➔ (*b*) Trails A	-.108	.512	-1.582	-1.818, .199
	SES ➔ (*c'*) Trails A	.617	.019	4.478	.049, .125***
	**Indirect Effect (*ab*)**	**.040**	**.035**[b]		**-.009, .136**
Model 6d					
	SES➔ (*c*) Stroop Words	-.236	2.025	-1.285	-6.611, -1.406
	SES ➔ (*a*) EC	-.450	.003	-2.539	-.015, -.002*
	EC ➔ (*b*) Stroop Words	.069	57.394	.733	-71.548, 155.723
	SES ➔ (*c'*)Stroop Words	-.205	2.082	-1.085	-6.381, 1.863
	**Indirect Effect (*ab*)**	**-.031**	**.045**[b]		**-.149, .035**

c is the total effect of SES on the relevant outcome variable; the path coefficients (*a*, *b, c'*) estimate the strength of hypothesized causal associations; *c'* is the direct effect of SES on the relevant outcome variable; *ab* estimates the strength of the indirect effect of SES on the relevant outcome variable through the mediating tract. There are no t- or p-values produced for the indirect effects. All coefficients are standardized; indirect effects are partially standardized because the predictor variable is dichotomous. All models are adjusted for age. ALIC = anterior limb of the internal capsule; EC = external capsule. [a]Confidence intervals did not cross zero; [b]Bootstrapping based standard errors. *$p < .05$; **$p < .01$; ***$p<.001$

Supplementary Table 4. SES by Age Moderated Mediation Models

	Path	*B*	*SE*	*t*	95% CI
Model 1e					
	SES ➔ (a_1) ALIC	-.018	.010	-1.731	-.038, .003
	Age (a_2) ➔ALIC	-.008	.004	-1.773	-.017, .001
	SESxAge (a_3) ➔ALIC	.006	.007	.882	-.007, .019
	ALIC ➔ (*b*) Trails B	-2.968	.971	-3.058	-4.882, -1.054**
	SES ➔ (*c'*) Trails B	.085	.044	1.912	-.003, .172
	**Moderated Mediation Index**	**-.017**	**.021**[b]		**-.071, .015**
Model 4e					
	SES ➔ (a_1) EC	-.011	.008	-1.269	-.027, .006
	Age (a_2) ➔EC	-.010	.004	-2.642	-.017, -.003**
	SESxAge (a_3) ➔EC	.003	.005	.436	-.008, .013
	EC ➔ (*b*) Trails B	-3.400	1.182	-2.875	-5.731, -1.068**
	SES ➔ (*c'*) Trails B	.091	.044	2.045	.003, .178*
	**Moderated Mediation Index**	**-.008**	**.018**[b]		**-.051, .024**
Model 6e					
	SES ➔ (a_1) EC	-.013	.008	-1.559	-.030, .004
	Age (a_2) ➔EC	-.011	.004	-3.008	-.018, .-004**
	SESxAge (a_3) ➔EC	.005	.005	.909	-.006, .016
	EC ➔ (*b*) Stroop	103.546	39.550	2.618	25.505, 181.586**
	SES ➔ (*c'*) Stroop	-3.867	1.459	-2.651	-6.746, -.989**
	**Moderated Mediation Index**	**.508**	**.583**[b]		**-.329, 2.120**

The path coefficients (a_1, a_2, a_3, b, c') estimate the strength of hypothesized causal associations; c' is the direct effect of SES on the relevant outcome variable. The moderated mediation index is the difference between conditional indirect effects. There are no t- or p-values produced for the indirect effects. All coefficients are unstandardized; in PROCESS, standardized coefficients are not available for models with moderators. ALIC = anterior limb of the internal capsule; EC = external capsule. [b]Bootstrapping based standard errors. *p < .05; **p < .01; ***p<.001

Supplementary Table 5. Mediation Models with Additional Cardiovascular Adjustments

	Path	*B*	*SE*	*t*	95% CI
Hypertension					
Model 1f					
	SES ➔ (*c*) Trails B	.501	.044	3.624	.072, .245***
	SES ➔ (*a*) ALIC	-.481	.003	-3.426	-.017, -.005***
	ALIC ➔ (*b*) Trails B	-.146	.972	-2.080	-3.939, -.104*
	SES ➔ (*c'*) Trails B	.431	.045	3.053	.048, .225**
	**Indirect Effect (*ab*)**	**.070**	**.037**[b]		**.011, .158**[a]
Model 4f					
	SES ➔ (*c*) Trails B	.501	.044	3.624	.072, .245***
	SES ➔ (*a*) EC	-.416	.003	-2.935	-.013, -.003**
	EC ➔ (*b*) Trails B	-.130	1.179	-1.871	-4.531, .119
	SES ➔ (*c'*) Trails B	.447	.045	3.183	.054, .229***
	**Indirect Effect (*ab*)**	**.054**	**.034**[b]		**.003, .134** [a]
Model 6f					
	SES ➔ (*c*) Stroop	-.536	1.473	-3.672	-8.316, -2.502***
	SES ➔ (*a*) EC	-.373	.003	-2.526	-.012, -.002*
	EC ➔ (*b*) Stroop	.140	40.319	1.900	-2.946, 156.184
	SES ➔ (*c'*) Stroop	-.484	1.488	-3.282	-7.821, -1.947**
	**Indirect Effect (*ab*)**	**-.052**	**.037**[b]		**-.150, .002**
Diabetes					
Model 1g					
	SES ➔ (*c*) Trails B	.479	.044	3.448	.065, .239***
	SES ➔ (*a*) ALIC	-.466	.003	-3.323	-.017, -.004**
	ALIC ➔ (*b*) Trails B	-.165	.974	-2.343	-4.206, -.362*
	SES ➔ (*c'*) Trails B	.402	.045	2.848	.039, .216*
	**Indirect Effect (*ab*)**	**.077**	**.039**[b]		**.017, .173**[a]
Model 4g					
	SES ➔ (*c*) Trails B	.479	.044	3.448	.065, .239***
	SES ➔ (*a*) EC	-.390	.003	-2.734	-.013, -.002**
	EC ➔ (*b*) Trails B	-.145	1.173	-2.089	-4.765, -.137*
	SES ➔ (*c'*) Trails B	.422	.045	3.010	.046, .222**
	**Indirect Effect (*ab*)**	**.057**	**.033**[b]		**.007, .142**[a]
Model 6g					
	SES ➔ (*c*) Stroop	-.535	1.457	-3.706	-8.275, -2.524***

	SES ➔ (*a*) EC	-.337	.003	-2.282	-.012, -.001*
	EC ➔ (*b*) Stroop	.134	39.874	1.851	-4.878, 152.493
	SES ➔ (*c'*) Stroop	-.490	498	-3.366	-7.840, -2.045***
	**Indirect Effect (*ab*)**	**-.045**	**.034**[b]		**-.138, .004**
Body Mass Index					
Model 1h					
	SES ➔ (*c*) Trails B	.471	.044	3.386	.062, .236***
	SES ➔ (*a*) ALIC	-.444	.003	-3.115	-.017, -.004**
	ALIC ➔ (*b*) Trails B	-.165	.958	-2.392	-4.181, -.402*
	SES ➔ (*c'*) Trails B	.397	.045	2.823	.038, .214**
	**Indirect Effect (*ab*)**	**.074**	**.037**[b]		**.015, .165**[a]
Model 4h					
	SES ➔ (*c*) Trails B	.471	.044	3.386	.062, .236***
	SES ➔ (*a*) EC	-.391	.003	-2.738	-.013, -.002**
	EC ➔ (*b*) Trails B	-.147	1.173	-2.121	-4.800, -.174*
	SES ➔ (*c'*) Trails B	.413	.045	2.944	.043, .219**
	**Indirect Effect (*ab*)**	**.057**	**.034**[b]		**.008, .147** [a]
Model 6h					
	SES ➔ (*c*) Stroop	-.555	1.458	-3.845	-8.482, -2.729***
	SES ➔ (*a*) EC	-.337	.003	-2.262	-.012, -.001*
	EC ➔ (*b*) Stroop	.136	39.509	1.887	-3.407, 152.526
	SES ➔ (*c'*) Stroop	-.510	1.468	-3.504	-8.040, -2.246***
	**Indirect Effect (*ab*)**	**-.046**	**.033**[b]		**-.133, .003**

c is the total effect of SES on the relevant outcome variable; the path coefficients (*a*, *b, c'*) estimate the strength of hypothesized causal associations; *c'* is the direct effect of SES on the relevant outcome variable; *ab* estimates the strength of the indirect effect of SES on the relevant outcome variable through the mediating tract. There are no t- or p-values produced for the indirect effects. All coefficients are standardized; indirect effects are partially standardized because the predictor variable is dichotomous. All models are also adjusted for age. ALIC = anterior limb of the internal capsule; EC = external capsule. [a]Confidence intervals did not cross zero; [b]Bootstrapping based standard errors. *$p < .05$; **$p < .01$; ***$p<.001$
